# Obituary

**Published:** 1891-12

**Authors:** J. Taft, Geo. W. McElhaney, L. Jack, F. Abbott, E. S. Talbot


### Obituary.

Died, at his home in Philadelphia, May 27th, 1891, of heart failure, James W. White, M.D., D.D.S., A.M., in the sixty-fifth year of his age.

Although he had been annoyed by different attacks of rheumatic pharyngitis for several years, his death was sudden and unexpected. While the years he lived were not many in number, his life, as numbered in deeds, was one of the longest. And it lessens the shock and pain on his removal, to know that he was spared a bed of pain and suffering.

Dr. White was born in 1826; descended on both sides from good old family stock. His father dying when he was only four years old, his educational advantages were few, and he was truly a self-educated man in the highest sense of the term. Whatever he read, whatever he learned, was labled and placed in his mind in an orderly manner, ready for use at an instant's notice. As a writer he was unexcelled for his faculty of condensation and his descriptive ability. His knowledge on any subject was exhaustive—whatever subject he pursued he learned it thoroughly, through and through. His reputation as a scientist and a physician was also of the highest. His contributions to dentistry were many and variable; he was the highest authority in the world on such subjects as diseases incident to dentition, and the relation the teeth bear to the entire organism.

But he was perhaps more widely known as editor of the *Dental Cosmos*, a position for which he was especially fitted. He did not regard a journal as a stand for the display of past and present events, merely a statement of what had occurred; but he viewed it in the light of a teacher, which should direct the minds of the readers to a higher plane that was always advancing higher, thus stimulating the readers to renewed energy.

His connections with the S. S. White Dental Manf'g Co., were of long standing; he was present at its beginning and watched and helped it grow until it became the largest company of its kind in the world.

He was a man of unusually well-rounded principles; liberal in all subjects, professional, religious and social. He was identified with many philanthropic movements, giving his time, his talents, his money willingly, cheerfully and unassumingly.

Many will mourn his death—the poor and oppressed, for they have lost a benefactor; the medical profession, for it has lost one of its brightest members; humanity, because a great and good man has gone. But a life such as his serves as a stimulus to all others to do what he has done, to be what he has been.

While not a member of this society the late Dr. James W. White was one of its constant visitors. His genial and kindly ways, his wise counsel and business qualifications were frequently called into requisition to aid in disposing of many trying questions.

And by his death this society has lost one of its best friends.

J. Taft,
Geo. W. McElhaney,
L. Jack,
F. Abbott,
E. S. Talbot.

Whereas, Death has removed from this association Dr. C. W. Lewis.

Whereas, He was one of its youngest members, full of promise, of a genial, cheering disposition which had endeared him to those who knew him.

*Resolved*, That we desire to place on the records of this association this memorial of our appreciation of his many good qualities and acknowledge our humble submission to the Divine will.

J. Taft,
Geo. W. McElhaney,
L. Jack,
F. Abbott,
E. S. Talbot.

